# Depletion of plasma membrane–associated phosphoinositides mimics inhibition of TRPM7 channels by cytosolic Mg^2+^, spermine, and pH

**DOI:** 10.1074/jbc.RA118.004066

**Published:** 2018-10-10

**Authors:** Tetyana Zhelay, Krystyna B. Wieczerzak, Pavani Beesetty, Gerald M. Alter, Masayuki Matsushita, J. Ashot Kozak

**Affiliations:** From the Departments of ‡ Neuroscience, Cell Biology, and Physiology and; § Biochemistry and Molecular Biology, Wright State University, Dayton, Ohio 45435 and; the ¶ Department of Molecular and Cellular Physiology, Graduate School of Medicine, University of the Ryukyus, Okinawa 903-0215, Japan

**Keywords:** channel activation, phosphoinositide, polyamine, magnesium, transfection, transient receptor potential channels (TRP channels), mutant, gain-of-function mutation, TRPM, voltage-sensitive phosphatase

## Abstract

Transient receptor potential cation channel subfamily M member 7 (TRPM7) is an ion channel/protein kinase belonging to the TRP melastatin and eEF2 kinase families. Under physiological conditions, most native TRPM7 channels are inhibited by cytoplasmic Mg^2+^, protons, and polyamines. Currents through these channels (I_TRPM7_) are robustly potentiated when the cell interior is exchanged with low Mg^2+^-containing buffers. I_TRPM7_ is also potentiated by phosphatidyl inositol bisphosphate (PI(4,5)P_2_) and suppressed by its hydrolysis. Here we characterized internal Mg^2+^- and pH-mediated inhibition of TRPM7 channels in HEK293 cells overexpressing WT voltage-sensing phospholipid phosphatase (VSP) or its catalytically inactive variant VSP-C363S. VSP-mediated depletion of membrane phosphoinositides significantly increased channel sensitivity to Mg^2+^ and pH. Proton concentrations that were too low to inhibit I_TRPM7_ when the VSP-C363S variant was expressed (pH 8.2) became inhibitory in WT VSP–expressing cells. At pH 6.5, protons inhibited I_TRPM7_ both in WT and VSP C363S–expressing cells but with a faster time course in the WT VSP–expressing cells. Inhibition by 150 μm Mg^2+^ was also significantly faster in the WT VSP–expressing cells. Cellular PI(4,5)P_2_ depletion increased the sensitivity of TRPM7 channels to the inhibitor 2-aminoethyl diphenyl borinate, which acidifies the cytosol. Single substitutions at Ser-1107 of TRPM7, reducing its sensitivity to Mg^2+^, also decreased its inhibition by spermine and acidic pH. Furthermore, these channel variants were markedly less sensitive to VSP-mediated PI(4,5)P_2_ depletion than the WT. We conclude that the internal Mg^2+^-, polyamine-, and pH-mediated inhibition of TRPM7 channels is not direct but, rather, reflects electrostatic screening and resultant disruption of PI(4,5)P_2_–channel interactions.

## Introduction

TRPM7 (channel kinase 1 (ChaK1)) and the closely related TRPM6 (channel kinase 2 (ChaK2)) are unusual membrane proteins containing a transient receptor potential (TRP)^3^ channel and a serine/threonine kinase domain (1–4). TRPM7 is highly expressed in cells involved in immunity (such as lymphocytes, macrophages, microglia, and mast cells), various cancer cell lines (5–11), as well as cardiac and vascular smooth muscle and the kidney (12–15). The commonly used cell lines HEK293, COS7, CHO-K1, HeLa, and others express substantial outwardly rectifying cation currents mediated by TRPM7 (16–19). A salient feature of TRPM7 channels is their sensitivity to cytoplasmic Mg^2+^, protons, and polyamines (6, 7). Mg^2+^ inhibits Jurkat T cell TRPM7 channels with a biphasic concentration dependence involving ∼10 μm and ∼165 μm IC_50_ inhibitor sites (20). Biphasic Mg^2+^ dependence has also been described for heterologously expressed murine TRPM7 channels (21). By contrast, intracellular protons inhibit with a single pKa of 6.3 (20). The mechanism of inhibition by these cations has remained a mystery since it was first described. We showed that Mg^2+^ is not unique in its inhibitory actions and that other divalent (Mn^2+^, Ca^2+^, Ba^2+^) and trivalent (La^3+^) metal cations inhibit in a similar fashion (22). The polyamines spermine (+4 charge), spermidine (+3), and putrescine (+2) inhibit native TRPM7 channels with potencies matching their overall positive electrostatic charge. Neomycin and poly-lysine, large polyvalent cations known to sequester anionic phospholipids (23), are also effective inhibitors (7). An alternative view proposes that inhibition of channel activity by Mg^2+^ reflects binding of Mg-ATP and other Mg-nucleotides to the C-terminal kinase domain, which somehow communicates this event to the pore and closes it (24–27). In support of this mechanism, channel Mg^2+^ sensitivity is reduced in cells from TRPM7 kinase–inactivated transgenic mice compared with the WT (28).

In whole-cell recordings, internal protons and polyvalent cations reduce TRPM7 current amplitude without altering its voltage dependence (6). By contrast, Mg^2+^ and polyamines applied on the extracellular side produce a rapid voltage-dependent channel pore block (6, 29, 30). Tonic blockade of inward monovalent current by external Ca^2+^/Mg^2+^ gives rise to the characteristic steep outward rectification of I_TRPM7_ (22).

At the single-channel level, inhibition by Mg^2+^ is a combination of two effects: a gradual reduction of the number of conducting channels and an abrupt ∼20% drop in the unitary monovalent (39 pS) conductance, seen only with higher Mg^2+^ concentrations (>100 μm) (31). In some patches, TRPM7 channels were not sensitive to low micromolar Mg^2+^, whereas in others, Mg^2+^ sensitivity increased upon repeated Mg^2+^ additions, a phenomenon we termed use dependence (*i.e.* sensitization). We hypothesized that, in whole-cell recordings, current rundown in the presence of Mg^2+^ reflects a gradual increase in the channels' sensitivity to Mg^2+^, akin to the sensitization observed in cell-free patches (31). Current rundown follows the depletion of phosphoinositides in the channel vicinity when ATP is absent (*e.g.* (6, 32)) and can be prevented simply by reducing the Mg^2+^ concentration to nanomolar levels without supplying exogenous phospholipids (7). Presumably, the role of ATP here is to enable replenishment of PIPs by endogenous phospholipid kinases (33–36). Rundown is commonly seen when micromolar or higher concentrations of Mg^2+^ or spermine are present in the internal solutions (7).

Depletion of membrane PI(4,5)P_2_ (hereafter referred to as PIP_2_) by phospholipase C β and γ inhibits whereas exogenous PIP_2_ activates TRPM7 channels (7, 32, 34). Expression of a heterologous protein that dephosphorylates plasma membrane PIPs at the 5′ position, the voltage-sensing phosphatase (VSP) (37, 38), suppressed TRPM7 channel activity (39).

We proposed previously that inhibition by high internal Mg^2+^, polyamines, and acidic pH represents screening (electrostatic shielding) of negative charges on the phospholipid co-factors of these channels without directly demonstrating this (7). Here we investigated whether depletion of PIP_2_ by expressing VSP is sufficient to mimic inhibition of TRPM7 channels by these cytosolic cations. We find that PIP_2_ depletion significantly increases the sensitivity of TRPM7 channels to Mg^2+^ and protons, in agreement with our hypothesis that these ions act by screening the negative charges of PIP_2_ phosphates. Sensitivity to propionate or 2-aminoethyl diphenyl borinate (2-APB), an inhibitor that acidifies the cytosol (40), is also significantly augmented by PIP_2_ depletion. TRPM7 Ser-1107 (41) mutants, which have been reported to be Mg^2+^-insensitive, were also less sensitive to spermine and pH. Importantly, the same mutants (S1107E and S1107R) were significantly less sensitive to PIP_2_ depletion than WT channels. These observations revealed that inhibition by internal Mg^2+^ and other cations shares a common mechanism and depends on cellular PIP_2_ levels.

## Results

### Effect of VSP expression on Mg^2+^ sensitivity of native TRPM7 channels

**Figure 1.**
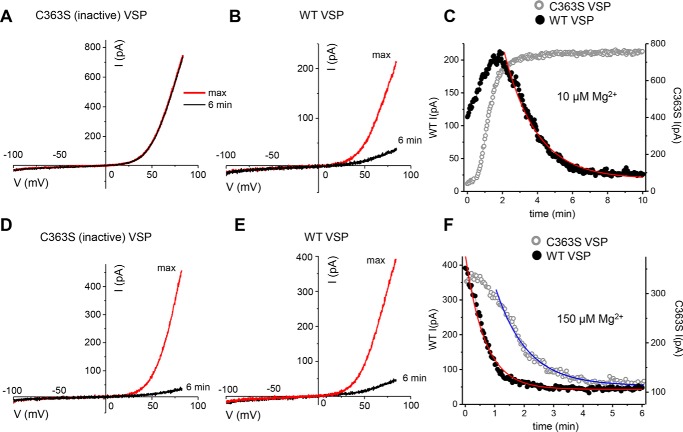
**Native TRPM7 channel currents in WT- and C363S VSP–expressing HEK cells.** *A*, *B*, *D*, and *E*, TRPM7 current–voltage relations with 10 μm (*A* and *B*) and 150 μm (*D* and *E*) free internal Mg^2+^ in WT (*B* and *E*) and C363S (*A* and *D*) CiVSP-expressing cells. *C* and *F*, time courses of current development and reduction with internal 10 μm and 150 μm Mg^2+^. In this and subsequent figures (except for Figs. 7 and 8), *filled* and *open symbols* represent current amplitudes measured in cells expressing WT and C363S VSP, respectively. The graphs in *A–F* were obtained from the same cells. The declining current amplitude was fitted with a single exponential decay function (*C* and *F*). The membrane potential between ramps was held at −60 mV.

HEK293 cells express significant magnesium-inhibited cation currents representing TRPM7 channel activity (20, 30). We took advantage of the ease of transfecting this cell type to investigate the effects of VSP-mediated PIP_2_ depletion on endogenous TRPM7 channel activity. We compared TRPM7 channel currents in HEK cells transfected with WT (active) and C363S mutant (inactive) CiVSP (38, 42). Fig. 1 shows current–voltage (I–V) relations obtained with 10 μm and 150 μm free [Mg^2+^]*_i_* in cells expressing WT and C363S VSP. I–V shapes were unchanged by VSP expression or by Mg^2+^ (Fig. 1, *A*, *B*, *D*, and *E*). In GFP+ cells transfected with C363S, there was no noticeable current reduction over time with 10 μm Mg^2+^ (Fig. 1*A*), whereas in WT VSP–expressing cells, the current declined during the experiment (Fig. 1, *B* and *C*).

**Figure 2.**
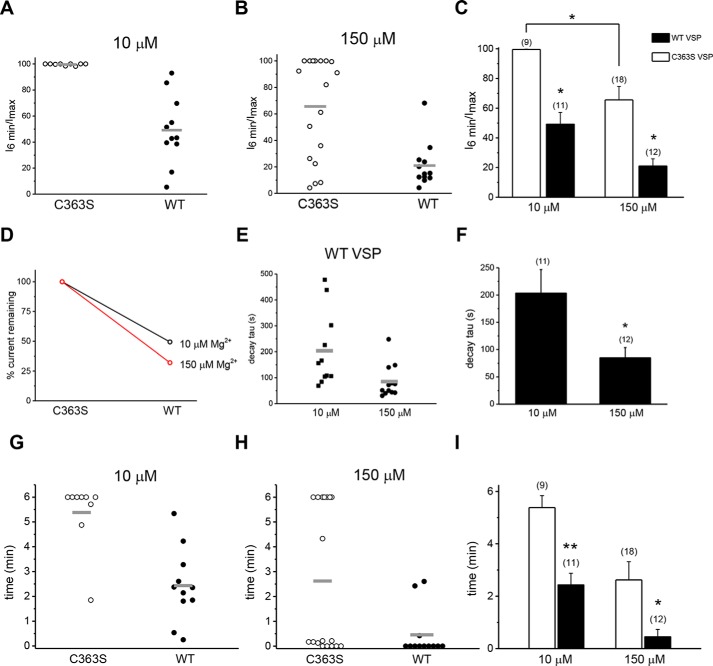
**Mg^2+^ dependence of native TRPM7 current amplitude and decay rate in VSP-expressing cells.** *A* and *B*, amplitudes measured in a whole cell at 6 min were divided by maximum attained current, set as 100% in each cell, and presented as percentages. Each point represents a ratio of measurements obtained from a single cell. Internal Mg^2+^ concentrations are shown in the graph. For cells in which the current continued to increase at and after 6 min, amplitudes at t = 6 min were taken as the maximum. *C*, summary of the experiments in *A* and *B*. For the indicated Mg^2+^ concentrations, the mean I_6 min_/I_max_ ratio was significantly lower for WT- than for C363S VSP–transfected HEK cells. *Empty* and *filled columns* here and in other figures represent data from C363S- and WT VSP–expressing cells, respectively. *, *p* < 0.002; Tukey's multiple comparisons test. Two C363S VSP–expressing cells in which decay was linear were excluded from the analysis. *D*, the data shown in *C* represented as a percentage of TRPM7 current remaining at 6 min in WT- relative to C363S VSP–expressing (set as 100%) cells, with free internal Mg^2+^ of 10 μm (*black*) or 150 μm (*red*). *E*, scatter graph of exponential decay time constant (τ) values in WT VSP–transfected cells at 10 and 150 μm Mg^2+^ concentrations. *F*, summary of the data shown in *E*. *, *p* < 0.05. *G* and *H*, times of whole-cell dialysis required to reach maximum current amplitude at the indicated Mg^2+^ concentrations. Each point represents a measurement obtained from a single cell. *I*, summary of the experiments in *G* and *H*. Mean rise times were shorter for WT- than for C363S VSP–transfected HEK cells for 10 μm and 150 μm [Mg^2+^]. Time = 0 represents cells that did not show a current increase after break-in. *, *p* < 0.05, **, *p* < 0.001 (*t* test). *Horizontal lines* in *C*, *F*, and *I* represent arithmetic means.

150 μm [Mg^2+^], by contrast, was sufficient to inhibit TRPM7 channels in all tested WT cells and the majority of C363S-expressing cells (Fig. 1, *D–F*). Time courses for 10 μm and 150 μm internal Mg^2+^ are shown in Fig. 1, *C* and *F*; in the absence of PIP_2_ depletion (*i.e.* C363S expression), currents developed slowly after the whole-cell configuration was established and usually reached a maximum 2–6 min later (addressed in more detail in Fig. 2). Current suppression by Mg^2+^ could be well-fitted with single exponential decay functions (Fig. 1, *C* and *F*).

We further analyzed the primary data presented in Fig. 1 by dividing the current measured at 6 min by the maximum attained amplitude for each cell. This ratio gives a simple measure of Mg^2+^-induced current decay that follows the initial rise phase (18). It is a single-cell, “steady state” alternative to population measurements of I_max_ commonly used in studies of TRPM7 channel Mg^2+^ dependence (*e.g.* (20, 24). In Fig. 2, *A* and *B*, I_6min_/I_max_ ratios for individual cells at two Mg^2+^ concentrations are presented, grouped according to the VSP variant expressed. These data, summarized in Fig. 2*C*, demonstrate that when PIP_2_ was not depleted (C363S VSP transfection), TRPM7 currents did not decay noticeably at 10 μm Mg^2+^ during our recordings. By contrast, expression of WT VSP and consequent PIP_2_ depletion resulted in a drastic sensitization of the channels to Mg^2+^; ∼50.8% to 61.1% of the maximum current declined after 6 min of dialysis. WT VSP–expressing cells that showed less decay most likely represent a subpopulation where the enzyme activity was low, leaving PIP_2_ levels high (Fig. 2*A*). Alternatively, these cells may express high levels of endogenous PI 5-kinase enzymes thought to be responsible for PIP_2_ replenishment (36).

The higher 150 μm Mg^2+^ concentration was sufficient to decrease currents in the majority of C363S-expressing cells (Fig. 2*B*). The decrease was more pronounced in WT VSP–expressing cells; on average, 78.9% of the current compared with 34.4% for C363S (Fig. 2, *B* and *C*). In a minority of C363S-expressing cells, 150 μm was ineffective, producing no current reduction, whereas such an Mg^2+^-insensitive population was absent among WT VSP–expressing cells (Fig. 2*B*). In WT VSP–expressing cells, percent remaining current was indeed higher at 10 μm (Fig. 2*D*, *black*) than 150 μm Mg^2+^ (Fig. 2*D*, *red*), suggesting that current decay is faster at higher Mg^2+^ concentrations.

WT VSP–expressing cells showed highly variable current amplitudes, even in the same batch of cells (see scatterplots in Fig. 2*A*). This variability may be due to the heterogeneity of HEK cell resting membrane potentials and consequent differences in basal lipid phosphatase activity during culturing (see “Discussion”). Because culturing, transfection, and voltage protocols were identical for all cells, it is unlikely that the degree of VSP activation during the recording was the cause of the observed disparity (36). An alternative explanation is that not all GFP+ cells express equal amounts of VSP because, in the transfected cell, GFP and VSP are synthesized from one transcript as separate proteins (see “Experimental Procedures”).

We next compared the decay phases of the current in WT VSP–expressing cells for both Mg^2+^ concentration (Fig. 1, *C* and *F*). The decay time constant (τ) was significantly lower with increased internal Mg^2+^ concentration (Fig. 2, *E* and *F*). Thus, at increased Mg^2+^ concentrations, the apparent onset of inhibition was accelerated in PIP_2_-depleted cells.

Exponential decay time constants for WT (*n* = 12 cells) and C363S-expressing cells (*n* = 8 cells) were compared for 150 μm Mg^2+^ but were not statistically significantly different (mean ± S.E. for WT and C363S were 85.13 ± 18.40 and 48.90 ± 5.81, respectively; Student's *t* test, *p* = 0.315).

We next focused our attention on the rising phase of TRPM7 current and its dependence on PIP_2_ levels. To this end, the times required to reach maximum were examined in WT- and C363S-expressing cells as a measure of the rate of current development after break-in using two Mg^2+^ concentrations. Unexpectedly, we found that rise times were shorter in PIP_2_-depleted cells than in control cells. Thus, at 10 μm Mg^2+^, the mean rise time was ∼6 min in control cells (C363S) but only ∼2 min in PIP_2_-depleted (WT) cells, a 3-fold change (Fig. 2, *G* and *I*). At 150 μm [Mg^2+^], the times to reach maximum were decreased in both C363S- and WT-expressing cells (Fig. 2*H*). This suggested that the effect of increasing [Mg^2+^] on the time to reach maximum current can also be mimicked by PIP_2_ depletion. The time to reach maximum current in each cell is set by a competition between the rising and the decay phase; *i.e.* when inhibition is accelerated, it will overwhelm the rising phase and manifest as an earlier drop in current magnitude.

To determine whether the shorter times to maximum in PIP_2_-depleted cells reflected a faster current activation, we fitted the rise times with monoexponential functions for C363S- and WT VSP–expressing cells. We limited our analysis to WT VSP–expressing cells that showed decay and C363S-expressing cells that showed no decay in current. At 10 μm Mg^2+^, the time constants were 94.01 ± 19.03 for C363S and 72.9 ± 12.36 for WT VSP (mean ± S.E.). Thus, there was a tendency for PIP_2_ depletion to accelerate the initial rise of current, which was not statistically significant (Student's *t* test). For 150 μm, such a comparison was not performed because the current did not increase in most WT VSP–expressing cells (Fig. 1). We conclude that the reduction in time to maximum (Fig. 2, *G–I*) is primarily due to an effect on the decay phase rather than the initial potentiation of the current.

### Effect of PIP_2_ depletion on pH regulation and 2-APB sensitivity of TRPM7 channels

**Figure 3.**
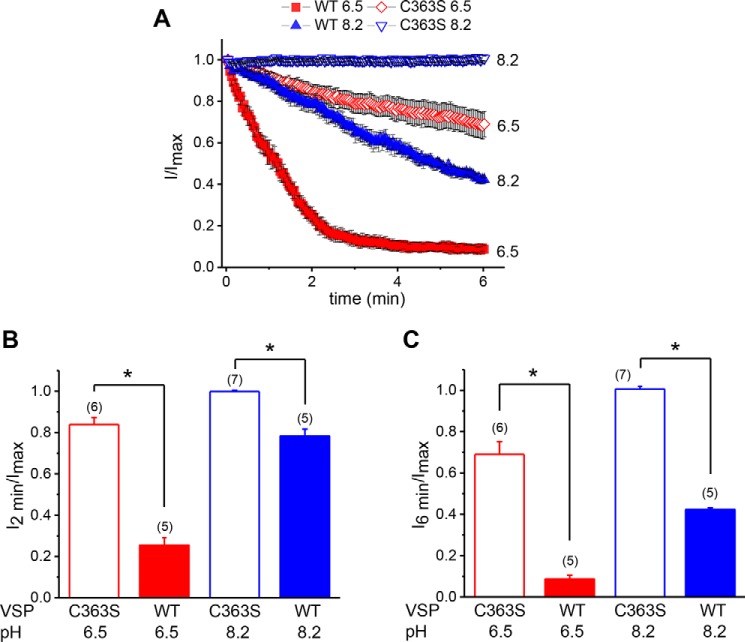
**pH dependence of native TRPM7 channels in WT- and C363S VSP–expressing cells.** *A*, time courses of TRPM7 current reduction obtained when the pH of the internal solution was 8.2 (*blue*) and 6.5 (*red*) in cells expressing WT (*filled*) and C363S VSP (*hollow*). Currents measured at +84.84 mV were normalized to I_max_ for each cell. Only the decay phase of the current is plotted against time. *B* and *C*, current inhibition at 2 min (*B*) and 6 min (*C*) for pH 8.2 and 6.5 obtained from *A*. *, *p* < 0.001. The exponential decay time constant (τ) for WT VSP at 6.5 was 76.17 ± 1.49 s, R^2^ = 0.99. Internal [Mg^2+^] was ∼80 nm in all recordings. The membrane potential was held at −60 mV between ramps.

Next we investigated whether the dependence of native TRPM7 channel activity on cytosolic pH is also governed by cellular PIP_2_ levels, as hypothesized previously (7, 20). We tested two internal solutions that were fixed at acidic (6.5) and basic (8.2) pH values. pH 8.2 (corresponding to [H^+^] ≈ 10 nm) did not suppress TRPM7 channels in control C363S cells but became inhibitory in WT VSP–transfected cells (Fig. 3) (20). In C363S-expressing cells, the TRPM7 channel current decayed slowly at pH 6.5 ([H^+^] ∼ 1 μm) (Fig. 3*A*). In WT VSP–expressing PIP_2_-depleted cells, the current reduction at pH 6.5 was markedly faster (Fig. 3, *A* and *B*). The internal free Mg^2+^ concentration in these experiments was held constant at 100 nm, a low concentration that prevents channel rundown (31, 40). 2 min or 6 min after reaching the maximum, remaining TRPM7 currents were significantly lower in WT VSP compared with C363S-expressing cells (Fig. 3, *B* and *C*).

A two-way analysis of variance performed using TRPM7 current amplitudes remaining 2 min after reaching maximum (Fig. 3*B*) showed a significant interaction between PIP_2_ depletion and the inhibitory effect of protons (*p* < 0.0001). Thus, decreasing the cytoplasmic pH had a higher inhibitory effect on TRPM7 channels that were PIP_2_-depleted (*i.e.* WT VSP–expressing cells) compared with nondepleted channels (C363S VSP).

**Figure 4.**
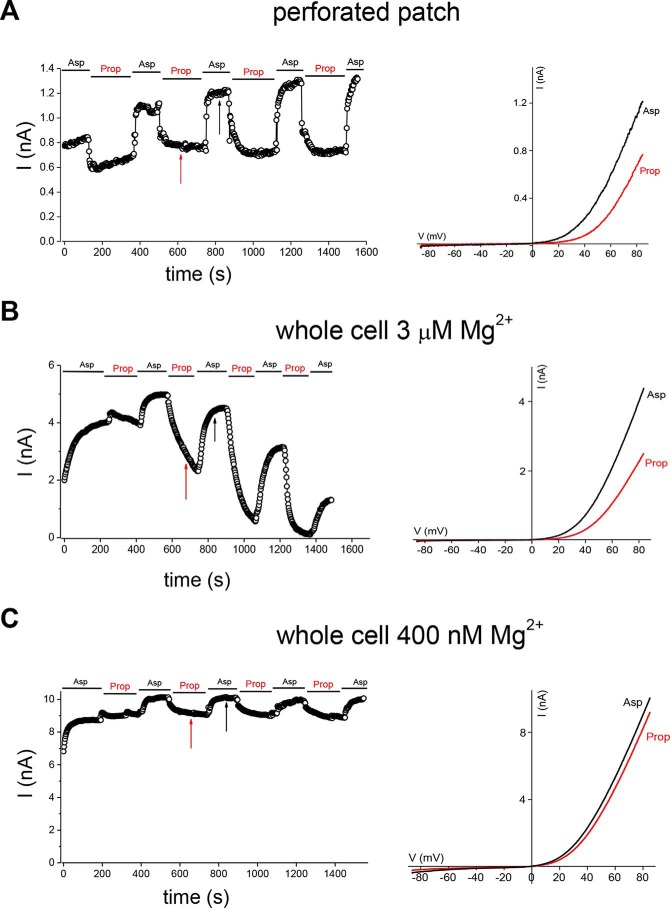
**Inhibition of recombinant TRPM7 channel currents by propionate-induced acidification in perforated patch and whole-cell configurations.** *A–C*, perforated-patch (*A*) and whole-cell (*B* and *C*) recordings of current inhibition by repeated application of 20 mm sodium propionate in HEK cells expressing WT mTRPM7-GFP. Time courses (*left panels*) and I-V relations (*right panels*) are depicted. *Vertical arrows* indicate the time points where I–Vs were obtained. Internal free [Mg^2+^] was 3 μm in *B* and 400 nm in *C*. Representative recordings were chosen from *n* = 5 (*A*), 5 (*B*), and 7 (*C*) experiments. The membrane potential between ramps was held at 0 mV.

To examine the effect of cytoplasmic acidification on TRPM7 channels in the absence of rundown, we employed the perforated patch recording configuration, which does not perturb the cellular Mg^2+^. In the perforated patch, TRPM7 channels do not run down, presumably because PIP_2_ is not depleted. We transfected HEK cells with mTRPM7 and studied its dependence on acidification induced by application of sodium propionate, a salt of a weak acid (7). As shown in Fig. 4*A*, repeated applications of 20 mm propionate resulted in reversible inhibition of TRPM7 currents. The extent of inhibition did not change significantly over time. We then performed the same experiment in whole-cell mode with 3 μm Mg^2+^ in the pipette (Fig. 4*B*). In this case, repeated application of 20 mm propionate resulted in progressively stronger inhibition of the current. 3 μm Mg^2+^ is sufficient to support TRPM7 current rundown, likely involving the high-affinity Mg^2+^ inhibitor site (20). In whole-cell recordings with a lower (400 nm) Mg^2+^ concentration, which does not support rundown, repeated applications of propionate inhibited the current to a similar extent (Fig. 4*C*). This behavior was similar to that seen in the perforated patch mode (Fig. 4*A*). Therefore, PIP_2_ depletion in whole-cell recordings results in stronger inhibition by cytoplasmic protons, which is not seen in perforated-patch recordings when PIP_2_ is not depleted. This is in agreement with the experiments described in Fig. 3, where PIP_2_ was depleted by VSP expression. Thus, the pH and Mg^2+^ sensitivities of TRPM7 channels are interdependent. The role of low micromolar Mg^2+^ in channel rundown only becomes apparent when PIP_2_ is depleted during whole-cell dialysis because the channels do not run down in the perforated patch even though the cytoplasmic Mg^2+^ is in the millimolar range.

**Figure 5.**
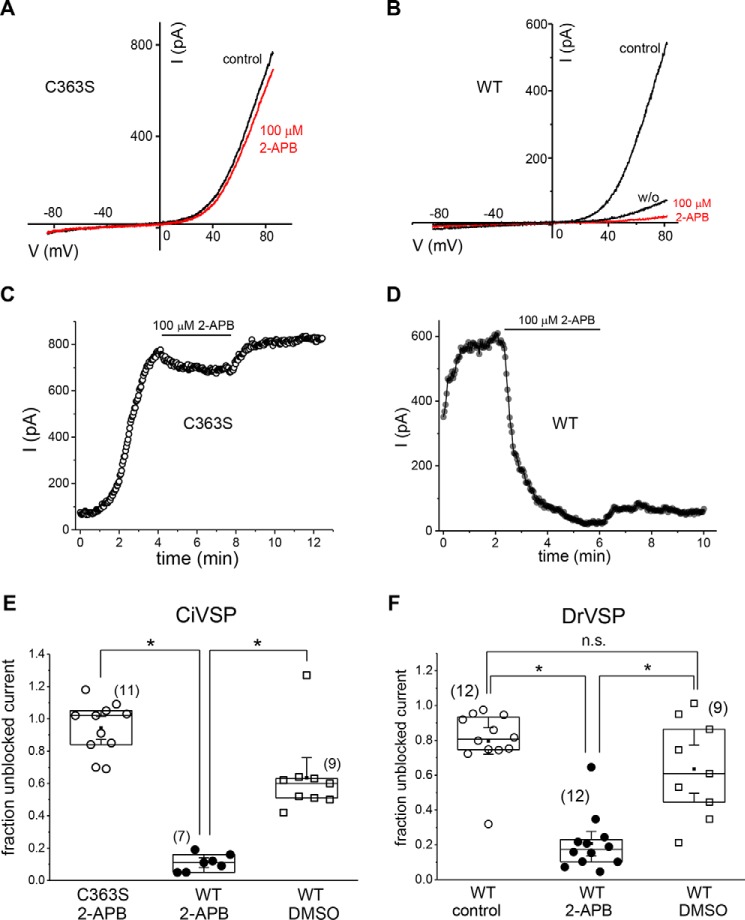
**Response of native I_TRPM7_ to 2-APB.** *A* and *B*, I–V relations in C363S- and WT CiVSP–expressing HEK cells immediately before (*black*) and in the presence of 100 μm 2-APB (*red*). The *w/o* trace in *B* refers to washout. *C* and *D*, corresponding time courses of I_TRPM7_ amplitude from the same cells as in *A* and *B* and 0.05% DMSO control. *E*, extent of current inhibition by 2-APB and 0.05% DMSO alone in C363S- and WT CiVSP–expressing cells. *F*, extent of current reduction in 100 μm 2-APB, 0.05% DMSO, and vehicle control in WT DrVSP–expressing cells. The fraction of unblocked current was obtained by dividing I_TRPM7_ amplitudes immediately before adding 2-APB or DMSO and after 72 (*E*) and 60 (*F*) voltage ramps in their presence. Y = 1 corresponds to no inhibition. *, *p* < 0.05; Student's two-sample *t* test. Internal free [Mg^2+^] was 400 nm, and the holding potential was 0 mV. *n.s.*, not significant.

2-APB, a compound widely used in ion channel research, inhibits TRPM7 channels indirectly by acidifying the cytosol (40). Therefore, we compared inhibition by 100 μm 2-APB in HEK cells to determine whether it depended on PIP_2_. In C363S CiVSP–expressing cells, this concentration of 2-APB had only a small effect on the current at internal free [Mg^2+^] of 400 nm (Fig. 5, *A* and *C*). By contrast, in WT CiVSP–expressing cells, 2-APB robustly inhibited the current in 1–2 min (Fig. 5, *B* and *D*). Washout of the drug fully reversed inhibition in C363S but only partially in some WT VSP–expressing cells (Fig. 5, *B* and *D*). Current decay in WT VSP–expressing cells was substantially slower when 0.05% DMSO was applied without 2-APB (Fig. 5*E*). On average, 2-APB inhibited 81.8% of the current for WT *versus* 8% for C363S cells, quantified ∼3.0 min after application (Fig. 5*E*). 2-APB inhibition was also increased in cells expressing WT DrVSP, which activates at more depolarized potentials than *Ciona* enzyme (Fig. 5*F*). As expected, 2-APB inhibition was voltage-independent (Fig. 5, *A* and *B*) (40). These results suggest that the potency of 2-APB is increased because TRPM7 channels in PIP_2_-depleted cells are more sensitive to inhibition by protons than in nondepleted controls.

### The effect of VSP on TRPM7 channels can occur without depolarization

**Figure 6.**
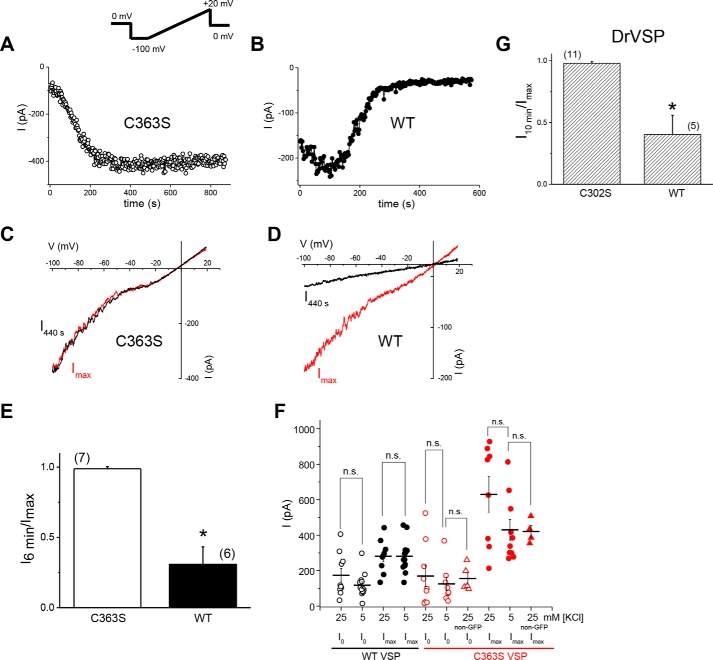
**Voltage dependence of VSP effect on native TRPM7 channels.** *A–D*, time courses of inward TRPM7 current (*A* and *B*) and I–V (*C* and *D*) in DVF Cs^+^-based bath solutions. Voltage ramps from −100 to +20 mV were applied. Data were obtained from HEK cells expressing WT or C363S CiVSP. *E*, current remaining after 6 min of whole-cell recording, divided by the maximum current attained in the respective cell. Inward current at −100 mV was plotted. Internal free [Mg^2+^] was 10 μm. *, *p* < 0.05. *F*, cells transfected with WT or C363S CiVSP were grown in RPMI 1640 medium containing 5.3 mm KCl or 25.3 mm KCl. Shown are TRPM7 current amplitudes at break-in and maximum currents attained. Current measurements taken from non-GFP cells among the C363S VSP–transfected cells were used as an untransfected control. Pairs grown in high and low K^+^ were not significantly different for WT and C363S VSP (*t* test, *p* > 0.05). [Mg^2+^]*_i_* = 400 nm. The holding potential was −60 mV. *n.s.*, not significant. *G*, TRPM7 current decay in C302S- and WT DrVSP–transfected cells. Shown is current remaining after 10 min of recording divided by the maximum current. −100 to +20 mV ramps were used as in *A–E*. The internal free [Mg^2+^] was 10 μm, and the external DVF solutions was Na^+^-based. The holding potential was 0 mV. *, *p* < 0.05.

TRPM7 and TRPM6 channel activity is commonly recorded by applying voltage ramps reaching +80 to +100 mV (see Fig. 1) (6). To rule out partial activation of VSP by depolarizations during voltage ramps that briefly reach +85 mV, as suggested for TRPM6 (39), we took advantage of the change in TRPM7 I–V relation when external divalent cations are removed; under such conditions, TRPM7 becomes semilinear and allows the measurement of large currents well below 0 mV, where CiVSP should be mostly inactive. We compared TRPM7 current decay using voltage ramps from −100 to +20 mV in WT and C363S VSP–expressing cells (Fig. 6, *A–D*). As seen with ramps reaching +85 mV (Fig. 1), native TRPM7 currents declined in minutes in WT but not C363S VSP–expressing cells (Fig. 6*E*). This observation is in agreement with reduced current magnitudes at break-in (I_0_) measured in WT VSP–expressing cells (Fig. S1, *A* and *C*) and suggests that VSP is active and can deplete PIPs in HEK cells even without depolarizing voltages.

HEK cells have reported membrane potentials of −40 to −50 mV (43). We therefore sought to evaluate VSP effects under depolarizing, high K^+^ conditions. Elevating the external [K^+^] from 5.3 mm of the normal RPMI medium to 25.3 mm is expected to shift the K^+^ equilibrium potential by +41 mV. Break-in current amplitudes in WT VSP-transfected cells grown in low (15 cells) and high [K^+^] (10 cells) that exhibited current decay were not significantly different (Student's test, *p* = 0.05) (Fig. 6*F*). We observed no differences in VSP-induced current decay in cells grown in 5.3 or 25.3 mm [K^+^] either (data not shown). The mean I_max_ was higher for C363S than WT VSP–expressing cells at both K^+^ concentrations (*p* < 0.05) (Fig. 6*F*). These results suggest that either the HEK cell membrane is depolarized already or there is significant lipid phosphatase activity that does not require depolarization. To address this question, we measured the resting membrane potentials of HEK cells in perforated patch recordings, obtaining mean ± S.D. values of −59.7 ± 2.1 mV, −47.5 ± 2.7 mV, −34.8 ± 1.7 mV, −32.4 ± 1.2 mV, −33.9 ± 2.4 mV, and −27.8 ± 0.9 mV (6 cells), similar to published values. Cells with more hyperpolarized membrane potentials exhibited a prominent outward K^+^ current upon switching to voltage clamp (data not shown).

Zebrafish VSP (DrVSP) is activated at more depolarized membrane potentials than its *Ciona* ortholog (44). The half-activation voltage of DrVSP is 94.27 ± 6.83 mV compared with 62.9 ± 4.5 mV for CiVSP (45). Expression of WT DrVSP in HEK cells resulted in a decay of native TRPM7 currents using −100 to +20 mV ramps, albeit at a slower rate (Fig. 6*G*). By contrast, currents did not decay in cells transfected with the inactive DrVSP C302S mutant (Fig. 6*G*). Collectively, the data presented in Figs. 6 and Fig. S1 (see also Fig. 5) strongly suggest that at least some VSP-induced PIP_2_ depletion occurs at negative membrane potentials and does not require depolarization.

### Point mutations reducing channel sensitivity to Mg^2+^ also reduce its sensitivity to spermine and pH

**Figure 7.**
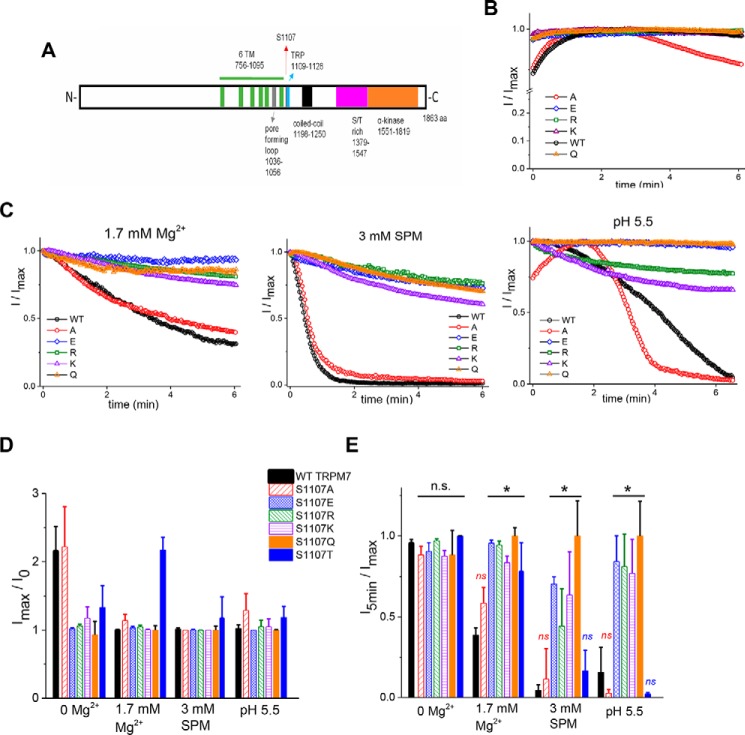
**Mg^2+^, spermine, and pH regulate TRPM7 channel activity through the same mechanism.** *A*, domain structure of TRPM7 with the position of Ser-1107 indicated by the *red arrow. B* and *C*, representative time courses of WT and S1107A, S1107E, S1107R, S1107K, and S1107Q mutant TRPM7 currents with internal 0 Mg^2+^ (*B*) and 3 mm total MgCl_2_ corresponding to calculated 1.7 mm free Mg^2+^, 3 mm spermine, and pH 5.5 (*C*). TRPM7 current was normalized to maximum current (I_max_) in each cell. *D*, mean maximum TRPM7 current amplitudes divided by break-in (I_0_) current in HEK cells expressing WT, S1107A, S1107E, S1107R, S1107K, S1107Q, or S1107T mTRPM7. *E*, bar graph showing mean current amplitudes at 5 min normalized to maximum current in each cell. The graphs in *D* and *E* were obtained from the same cells. The internal solution contained Mg^2+^, spermine, or an acidic pH of 5.5 as indicated. *, *p* < 0.05, Student's two-sample *t* test. * in *E* indicates significant differences in I_5min_/I_max_ compared with the WT at respective Mg^2+^, spermine, or proton concentrations. Experiments were performed with *n* = 4–11 cells for each condition. For five cells with 1.7 mm free internal Mg^2+^ and three cells with 3 mm spermine expressing S1107A, the command voltage ramps were applied every 2 instead of 2.5 s. The holding potential was 0 mV between ramps. *n.s.*, not significant.

Ser-1107 lies immediately outside of the TRP domain (Fig. 7*A*) of TRPM7 and has been reported to make Mg^2+^-insensitive ion channels when substituted with glutamate (41). This serine residue is conserved in the human TRPM7 and in the closely related TRPM6 channel. In view of our proposal that polyamines and pH exert inhibition of TRPM7 channels through the same mechanism as Mg^2+^ (see “Introduction”), we tested various S1107 mutants for their sensitivity to spermine and pH. We first tested the S1107E mutant, originally described as Mg^2+^-insensitive (41). Indeed, both spermine and protons were less inhibitory for this mutant (Fig. 7, *B–E*). We further examined the 1107 position by mutagenizing this serine to positively charged lysine and arginine instead of negatively charged glutamate. Both lysine and arginine mutants also exhibited diminished Mg^2+^ sensitivity accompanied by reduced spermine and pH sensitivity (Fig. 7, *B–E*). As a next step, we mutagenized the same serine to alanine, glutamine, threonine, and aspartate. Surprisingly, no correlation between the charge of this residue and channel sensitivity to Mg^2+^ was observed. However, we found that the size (surface area) of the substituting residue determined the channel phenotype. For example, changing Ser-1107 to glutamic acid or glutamine, having a similar size but differing in charge, both resulted in Mg^2+^-insensitive channels (summarized in Fig. 7*E*).

WT, S1107A, and S1107T TRPM7 magnitudes increased after break-in with 0 Mg^2+^ internal solution, whereas S1107E, S1107R, S1107K, and S1107Q currents were at their maximum magnitude at break-in (I_0_) and did not rise (Fig. 7, *B* and *D*). On the other hand, WT, S1107A, and S1107T mutants showed only very small current rises in the presence of internal 1.7 mm Mg^2+^, 3 mm spermine, and pH 5.5 (Fig. 7, *C* and *D*). This shows that the rising phase of TRPM7 current after break-in is due to the removal of inhibitory cations such as Mg^2+^ or polyamines and protons. Because S1107E, S1107R, S1107K, and S1107Q (gain-of-function (GOF) mutations) are insensitive to these inhibitory cations, they also do not show a rising phase with 0 internal Mg^2+^. The S1107C mutant failed to express substantial currents, whereas S1107D behaved as the other GOF mutants (data not shown). These experiments demonstrate that Mg^2+^ is not unique in its inhibition of TRPM7 and shares this property with other cations, such as polyamines and protons. Nevertheless, GOF mutations did not completely eliminate the Mg^2+^ sensitivity of TRPM7. At very high free Mg^2+^ concentrations (tested at 2.9 and 4.9 mm), both the S1107E and S1107K mutant channels were partially inhibited (data not shown).

### S1107 gain-of-function mutants are less sensitive to PIP_2_ depletion by VSP

**Figure 8.**
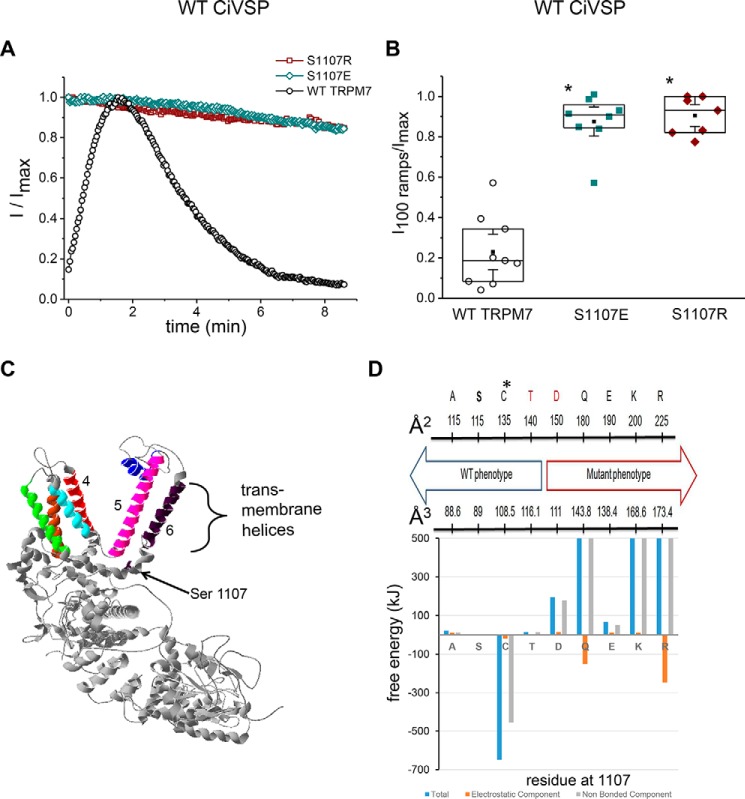
**Ser-1107 is involved in mediating TRPM7 sensitivity to PIP_2_ depletion by VSP.** *A*, representative time courses of WT, S1107E, and S1107R TRPM7 currents in cells co-transfected with WT CiVSP. The TRPM7 current at each time point was normalized to maximum current in the respective cell. Note that, unlike WT, for Glu and Arg mutants, the current does not increase after break-in. *B*, current amplitudes 100 ramps (about 4.2 min) after reaching maximum, divided by maximum current amplitude in HEK cells co-expressing WT VSP with WT, S1107E, or S1107R TRPM7. The internal solution contained 12 mm EGTA and 0 MgCl_2_. *, *p* < 0.05. Student's two-sample *t* test; significant difference compared with the WT. *C*, partial structural model of a TRPM7 monomer predicted as described under “Experimental procedures.” Transmembrane helices 1 through 6 are colored *brown*, *green*, *cyan*, *red*, *magenta*, and *dark purple*, respectively. The pore helix is shown in *blue*. The *arrow* indicates the position of residue Ser-1107. The cytoplasmic portion of the protein is shown in *gray. D*, summary of experimental findings from Ser-1107 mutagenesis. *Numbers* represent predicted surface areas (square angstroms, *top*) and volumes (cubic angstroms, *bottom*) of amino acid residues obtained from Refs. 67, 68. C* denotes the S1107C variant, which did not give rise to functional channels. Differences in threading energies between native and mutant TRPM7 models at locations within 8 Å of position 1107 are shown below. Electrostatic (*red bars*), non-bonding/steric (*gray*), and total (*blue*) energies were calculated as described under “Experimental procedures.”

As a logical next step, we investigated the behavior of mutants with diminished Mg^2+^, spermine, and pH sensitivity (GOF mutants) by co-expressing CiVSP. We reasoned that if Mg^2+^, spermine, and protons inhibit TRPM7 by screening PIP_2_-negative charges, then depleting membrane PIP_2_ would mimic this behavior. In other words, Mg^2+^-insensitive mutants may also be VSP-insensitive. Fig. 8*A* shows whole-cell recordings of WT TRPM7 and two GOF mutants with 0 Mg^2+^*_i_*. As expected, the WT current in VSP-expressing cells increased (because of cytosolic Mg^2+^ washout) and then decayed over time because of further PIP_2_ depletion. By contrast, S1107E and S1107R currents lacked a rising phase and decayed significantly less over the same time period (Fig. 8, *A* and *B*). This experiment demonstrated that channel sensitivity to Mg^2+^, polyamines, and protons strongly correlates with their sensitivity to PIP_2_ depletion, suggesting that they reflect the same process. To explore the sensitivity of TRPM7's local structure to changes at position 1107, a truncated model of TRPM7 was constructed and used to probe the consequences of changes at position 1107 (Fig. 8*C*). Replacement of serine with various residues examined here caused very modest solvent exposure changes, 1–4%, for residues within 8 Å of position 1107. This result was anticipated because the software we used finds only the structure's closest energy minimum and does not allow for the substantial structural changes necessary to locate a new global energy minimum. The degree of instability/stability introduced into the structure by each residue change was estimated by calculating the change in the force field free energy of the 1107 residue and neighboring residues (within 8 Å) relative to the native structure (Ser-1107). The results are shown in Fig. 8*D* (see Fig. S2 for the full scale), where the total free energy together with electrostatic and nonbonded components are plotted. The total and nonbonded free energies for the Gln (106,023 kJ and 106,174 kJ, respectively), Lys (2,445 kJ and 2,433 kJ), and Arg (11,455 kJ and 11,699 kJ) substitutions are very large and unfavorable, indicating an extremely large driving force for structural change. Other substitutions (Asp and Glu) are associated with smaller free energy changes but are also unfavorable and of substantial magnitude, indicating a driving force for structural changes in these mutants. Last, the electrostatic interactions are relatively small in magnitude. These results suggest that with Ala, Ser, Cys, or Thr at position 1107, the structure is stable whereas it is not with Asp, Gln, Lys, or Arg at the same position.

## Discussion

We have explored the relationship between well-known regulators of TRPM7 channels: membrane phosphoinositides on one hand and cytoplasmic Mg^2+^, spermine, and pH on the other. We employed expression of VSP to dephosphorylate the endogenous plasma membrane PIP_2_ and other phosphoinositide substrates, such as PI(3,4,5)P_3_ (46). Our main findings are as follows. Low Mg^2+^ and proton concentrations that were not inhibitory in inactive C363S VSP variant–expressing cells (10 μm Mg^2+^ and pH 8.2) significantly inhibited the native TRPM7 channel current when PIP_2_ was depleted by WT VSP activity. At higher concentrations, Mg^2+^ (150 μm) and protons (pH 6.5) were inhibitory in both WT- and C363S VSP–expressing cells but with a faster time course in the former. Native TRPM7 current inhibition by 2-APB was significantly more robust in WT *Ciona* or zebrafish VSP-expressing cells. Point mutations reducing TRPM7 channel sensitivity to Mg^2+^ also reduced its sensitivity to spermine and pH. Point mutants insensitive to these cations were also less sensitive to VSP co-expression.

Specifically, several parameters were changed by VSP expression: the extent of current reduction after the initial rise, measured at 5 or 6 min; the time constant of current decay; and the time to reach maximum. During whole-cell recording with low Mg^2+^, the TRPM7 current develops slowly, reaching maximum amplitude after several minutes of cell dialysis (Fig. 1, *A–C*). It is thought that this rise time reflects the removal of cytosolic Mg^2+^ (which likely occurs in seconds) and other downstream events. Whole-cell recordings (without VSP expression) with no Mg-ATP are accompanied by gradual PIP_2_ depletion (*e.g.* Refs. 32, 47). In perforated patch recording, which prevents Mg^2+^ and ATP loss (7, 48), heterologous TRPM7 channel currents were largely time-invariant (Fig. 4*A*).

Ionized [Mg^2+^] in mammalian cells is in the 0.5–1 mm range (49–51). In our view, the initial current development or potentiation represents Mg^2+^ (and proton) removal from PIP_2_ molecules that are bound to the channels in an intact cell because the average pKa of PIP_2_ is estimated to be near pH 6.3 (52). We found that the time to maximum was shortened in PIP_2_-depleted cells (Fig. 2). The difference between C363S and WT VSP-transfected cells was reduced at the high 150 μm Mg^2+^ concentration, reflecting the fact that this concentration is sufficient to inhibit TRPM7 even in cells not depleted of PIP_2_. High Mg^2+^, in effect, occludes PIP_2_ depletion. We also compared the initial current rise phase in C363S and WT VSP–expressing cells and found a small reduction in the time constant for WT VSP that was not statistically significant. We conclude from these measurements that the effect of PIP_2_ depletion on the rising phase of the current is primarily due to increasing sensitivity of the channels to Mg^2+^ and accelerated current decay. We do not rule out a direct effect on the initial rise speed, which could be due to a faster off-rate for Mg^2+^ when fewer PIP_2_ molecules are bound to the channel. Conversely, the Mg^2+^ on-rate, underlying current decline, is enhanced for the same reason; *i.e.* Mg^2+^ has to screen fewer PIP_2_ molecules to close the channels. In other words, the channels become more prone to rundown (31). Alternatively, after binding Mg^2+^, the PIP_2_–Mg^2+^ complex is dissociated from the channel, which might help explain why inhibition is slow, requiring several minutes for completion (Figs. 1 and 2).

Stimulation of Gα_q_-coupled phospholipase Cβ resulted in inhibition of TRPM7 channels through PIP_2_ hydrolysis *per se* because the downstream metabolites diacylglycerol and inositol trisphosphate had no effect (34). Application of water-soluble PIP_2_ analogs rescued rundown channel activity in that and other reports (7, 13, 34). In our hands, the expression of high levels of CiVSP resulted in significant reductions in both break-in and maximum current amplitudes (Fig. S1), supporting the view that PIP_2_ is a required cofactor for proper TRPM7 channel function.

We used VSP overexpression to take advantage of testing PIP_2_ dependence within the physiological range as opposed to applying exogenous water-soluble PIP_2_ analogs at very high concentrations. In some cases, exogenously applied PIP_2_ activated ion channels that were later found to be PIP_2_-insensitive when endogenous phospholipids were depleted by using genetically engineered tools (53, 54).

In WT VSP–expressing cells, the rates of Mg^2+^-dependent inhibition could be described by single exponential decay functions. We compared the decay time constants at the two Mg^2+^ concentrations and found that increasing the internal Mg^2+^ concentration to 150 μm resulted in significantly lower time constants (Fig. 2). We interpret this effect as faster overall on rate of Mg^2+^ when the number of PIP_2_ molecules is reduced by VSP. The rate of inhibition by an acidic pH of 6.5 was also substantially higher in PIP_2_-depleted cells (Fig. 3). Thus, depletion of PIP_2_ sensitized TRPM7 channels to these inhibitors.

We had to calibrate VSP expression levels to avoid greatly reduced TRPM7 currents (Fig. S1). Surprisingly, even without applying long depolarizing steps, which VSP is believed to require for activation (38), PIP_2_ levels were apparently lowered when culturing transfected cells for several days. This resulted in great variability of all parameters describing inhibition despite our efforts to keep transfection conditions identical in all cases. Such variability could arise from the differences in resting membrane potentials among HEK cells or differences in VSP expression levels. HEK293 cell resting membrane potentials have been estimated to be in the −50 to −30 mV range (55–58). To investigate whether depolarizing shifts in the resting membrane potential will increase VSP activity, transfected HEK cells were cultured in 25.3 mm KCl–containing RPMI medium for 24 h before recordings were made (Fig. 6*F*). For HEK cells expressing WT and C363S, the break-in and maximum current amplitudes in high and normal K^+^ (Fig. 6*F*) were not statistically significantly different (Student's *t* test). Our own measurements of HEK cell membrane potential in 4.5 mm [K^+^]*_o_* showed that it varied between −59 and −27 mV (see “Results”). This points to the existence of substantial VSP activity at these negative membrane potentials. Accordingly, it has been demonstrated previously that the CiVSP enzyme is active over a wide range of membrane potentials achievable in patch clamp experiments (42, 44).

We also looked for acute effects of VSP on TRPM7 channel currents by applying a long (2 s) depolarizing step either to +100 mV or to −10 mV and comparing the speed of current decay but saw no noticeable acceleration of current inhibition with +100 mV steps, a sufficient depolarization for VSP activation, used by other investigators for this purpose (*e.g.* Refs. 35, 53) (data not shown). It appears that, in the case of low-affinity interactions of PIP_2_ with channels, the basal activity of VSP is sufficient to account for the observed effects on Mg^2+^ and pH sensitivity. The command voltage ramp protocol applied in our experiments to measure TRPM7 currents briefly reaches +85 mV (see “Experimental procedures”) and may, by itself, be sufficient to activate VSP. This has been proposed for a similar voltage protocol (−120 to +100 mV applied at 1 Hz) used to record TRPM6 (39). Arguing against this scenario is our observation that high-efficiency VSP transfection reduced break-in TRPM7 currents for WT- compared with C363S VSP–expressing cells (Fig. S1).

In addition to PIP_2_, VSP can hydrolyze PI(3,4,5)P_3_ while generating PI(4)P in the process (46, 54, 59). Although participation of these phospholipids in TRPM7 channel regulation is not ruled out, most experimental evidence points to PIP_2_ as the primary anionic phospholipid required for TRPM7 activation (39).

Electrostatic interactions with PIP_2_ have been linked to increased Mg^2+^ sensitivity of several ion channels. Hydrolysis of PIP_2_ resulted in a greater degree and speed of TRPV5 channel inhibition by internal Mg^2+^ (60). Du *et al.* (61) showed that the Mg^2+^ and pH sensitivity of Kir channels depends on the availability of PIP_2_ and its interaction with the channels. Accordingly, point mutations strengthening Kir2.3 channel–PIP_2_ interactions reduced channel inhibition by Mg^2+^ and protons (compare with our Figs. 7 and 8), whereas those weakening Kir2.1-PIP_2_ interactions increased the Mg^2+^ and proton sensitivity. The authors proposed that the Mg^2+^ effect was mediated by stimulation of lipid phosphatases. A dominant-negative point mutation in Kir2.1 linked to Andersen syndrome decreased channels' PIP_2_ sensitivity, resulting in increased inhibition by Mg^2+^ (62). PIP_2_-sensitive KCNE-KCNQ channels are inhibited by intracellular Mg^2+^ and polyamines, and elevated PIP_2_ concentrations decreased inhibition by these cations (63). It can be summarized that low-affinity channel-PIP_2_ interactions are disrupted by physiological levels of Mg^2+^, protons, and polyamines, whereas high-affinity interactions are less sensitive to these cations (36, 54, 61). In agreement with this view, polyvalent cations with low charge-screening capacity, such as hexamethonium, do not inhibit TRPM7 (7).

We showed previously that inhibition of native TRPM7 channels by 2-APB is not direct but by cytoplasmic acidification induced by this drug (40). In PIP_2_-depleted cells, same concentration of 2-APB was significantly more potent than in controls (Fig. 5), in agreement with increased pH sensitivity of TRPM7 channels under these conditions (Fig. 3). Inhibition by 2-APB was considerably faster for WT than for C363S, consistent with a faster proton-induced I_TRPM7_ decline (Fig. 3). It would be instructive to test whether other TRPM7 modulators that depend on internal Mg^2+^ (64) also demonstrate increased potencies in PIP_2_-depleted cells.

Several basic residues have been implicated in PIP_2_ sensitivity of TRPM7 channels. Mutagenesis of basic Lys-1112, Arg-1115, and Lys-1125 (triple mutant) located in the TRP domain (Fig. 7*A*) to neutral glutamines resulted in nonfunctional channels (39). Because TRPM7 forms a homotetramer (4, 65), these results suggest that mutagenizing these three basic residues in all four subunits completely abolishes channel function. K1112Q TRPM7 (39), however, had low basal activity that could be potentiated by external NH_4_^+^ to alkalinize the cytosol and neutralize the inhibitory protons (7, 39). It is not known whether additional PIP_2_-interacting sites exist in TRPM7 protein and whether every subunit needs to bind PIP_2_ for the channel to open. For another tetrameric channel, binding to three subunits was sufficient to support channel activity (66). The effect of PIP_2_ depletion on TRPM7 channels may either represent multiple PIP_2_ binding sites on each subunit and/or removal of PIP_2_ from an increasing number of subunits comprising the channel. Further experimentation will be needed to answer this question directly.

Ser-1107 was described as a key residue mediating Mg^2+^ inhibition of TRPM7 (41) (Fig. 8*C*): substitution with negatively charged glutamate resulted in channels with greatly reduced Mg^2+^ sensitivity. We investigated what other amino acid substitutions in this position would reduce Mg^2+^ sensitivity (gain-of-function mutations). Surprisingly, substituting Ser-1107 with positively charged arginine or lysine resulted in an identical phenotype (Figs. 7 and 8*D*). Uncharged glutamine also gave rise to channels insensitive to Mg^2+^ (Figs. 7 and 8*D*). Substitution with alanine or threonine, on the other hand, resulted in channels that behaved like the WT. We then tested whether these point mutants were sensitive to other positively charged inhibitors such as spermine and protons, finding that the GOF mutants were strikingly less sensitive to both, whereas the S1107A and S1107T mutants were inhibited by all three, like the WT channel (Fig. 7).

We further tested S1107 mutants for their sensitivity to PIP_2_ depletion by co-expression with VSP. We found that S1107E and S1107R mutants were also significantly less sensitive to VSP-induced PIP_2_ depletion compared with the WT (Fig. 8, *A* and *B*). In combination, these results suggest that Ser-1107 is critical for the sensitivity of TRPM7 to cations, polyamines, and PIP_2_ interaction and that certain substitutions at this site can disrupt this interaction. Mutations by which PIP_2_–channel interactions are potentially strengthened (S1107E, S1107R, S1107K, and S1107Q) are also more difficult to disrupt by the screening cations. Indeed, we were able to inhibit GOF mutant currents only by raising [Mg^2+^] to 2.9 mm and higher (see “Results”). It is notable that the positive regulator of TRPM7 channel activity (PIP_2_) and the negative regulators (Mg^2+^, spermine, and protons) share the same site of action, suggesting that Mg^2+^, polyamines, and pH exert their inhibitory actions through the same mechanism: electrostatic screening (shielding) of the PIP_2_-negative charge (7). We therefore predict that neomycin and polylysine, polycations that efficiently interact with PIP_2_, will also be less potent in GOF mutants compared with the WT. We also predict that the S1107D, S1107K, and S1107Q mutants will behave like Glu and Arg, whereas S1107A and S1107T are likely to behave like WT channel in terms of VSP sensitivity. The phenotype of S1107 substitutions does not depend on the charge in that position because arginine, lysine, and glutamate behave similarly. Rather, it depends on the bulk as reflected in the surface area of the substituting amino acid residue (67, 68). Thus, alanine (surface area of 115 Å^2^) and threonine (140 Å^2^) mutants are similar to WT serine (115 Å^2^), whereas larger side chains (Asp, Gln, Glu, Lys, and Arg) with respective surface areas of 150, 180, 190, 200, and 225 Å^2^ make the channel insensitive to Mg^2+^ and other cations (Fig. 8*D*). Acidic glutamate (190 Å^2^) and uncharged glutamine (180 Å^2^) behave similarly, having a similar size. The cutoff appears somewhere between 140 and 150 Å^2^ (residues in *red* in Fig. 8*D*) because the threonine mutant behaved as the WT, whereas aspartate was a GOF mutant. Surprisingly, the exposure of residue side chains within the truncated TRPM7 model used here changes very little (1% to 4%) despite variations in the size of the mutant side chains at 1107 (Fig. S2). For some mutations at position 1107 (Gln, Lys, and Arg), energies are extremely large and unfavorable. Others (Asp and Glu) are more modest, but they are also unfavorable and of significant magnitude. This may provide the energetic driving force for a conformational change in the region of residue 1107 that modifies or abolishes PIP_2_ interactions with the protein. Contributions from electrostatic interactions are not as great as those from nonbonded (steric) interactions and are even stabilizing in the case of Gln and Arg. This suggests that the bulk of the side chains rather than their charge is of greatest consequence. In total, modeling supports functional analyses that indicate that Ala and Cys mutants retain native activity whereas Asp, Glu, Gln, Lys, and Arg do not (Figs. 7 and 8). These findings are unexpected because many publications until now have argued that PIP_2_ interacts with basic amino acids of the channel protein (*e.g.* Refs. 39, 54, 69–71). Apparently, the side chain bulk plays an important role in these interactions in the case of TRPM7. We are not aware of other studies implicating amino acid size as a determinant of PIP_2_ sensitivity of channels. Whether Ser-1107 directly binds PIP_2_ was not determined. It might, for example, function downstream of PIP_2_ binding by communicating with the channel gating machinery. It is unlikely to bind Mg^2+^ or other inhibitory cations, however.

Ser-1107 in TRPM7 or its corresponding Ser-1080 in human TRPM6 have not been identified as phosphorylation sites in intact cells so far, suggesting that they influence TRPM7 channel activity by a mechanism independent of TRPM7/6 kinase activity (72, 73).

In this paper, we demonstrate that PIP_2_ depletion can mimic the inhibitory effects of internal Mg^2+^ and pH on TRPM7 channel activity. Our interpretation of VSP data assumes that PI(4,5)P_2_ phospholipid is its main substrate. Several interesting questions about TRPM7 regulation can be addressed in our future experiments. What is the effect of PIP_2_ depletion at the single-channel level? Mg^2+^ inhibition consists of a gradual disappearance of conducting channels and a modest, ∼20% reduction in unitary conductance (31); will VSP-mediated PIP_2_ depletion mimic both of these effects? The latter effect is particularly interesting because it must involve the ion conduction pathway itself. It is also not known whether pH and polyamine effects on single-channel TRPM7 activity are similar to Mg^2+^ effects and whether the high-affinity Mg^2+^ inhibitory site is also PIP_2_-dependent. The question of the minimum subunit number required to bind PIP_2_ to activate the channel remains open and will allow a more quantitative description of channel inhibition by polyvalent cations.

## Experimental procedures

### Cell maintenance and transfection

The HEK293 cell line was maintained in RPMI 1640 medium (Lonza, Walkersville, MD) supplemented with 10% heat-inactivated fetal bovine serum (Fisher Scientific, Fair Lawn, NJ) and penicillin/streptomycin (HyClone) in a cell culture incubator (Forma Scientific, Marietta, OH) at 37 °C and 5% CO_2_ as described previously (20). Cells were grown in 10-cm polystyrene cell culture dishes (USA Scientific, Ocala, FL) and passaged twice a week. Cells plated in 6-well polystyrene plates (USA Scientific) the day before were transfected either with CiVSP or DrVSP complementary DNA in pIRES2-EGFP bicistronic plasmid vectors provided by Yasushi Okamura (Osaka University, Japan). Inactive C363S (*Ciona*) and C302S (zebrafish) variants were transfected as negative controls. Transient transfection of plasmid DNA was performed with TransIT-LT1 reagent (Mirus Bio, Madison, WI) according to the manufacturer's recommendations. 2–3 days after transfection, the cells were lifted and transferred to glass-bottom recording chambers. In one series of experiments, the transfection mixture was kept in the wells until the start of recordings (∼48 h) (Fig. S1), whereas in the other series, it was washed away by changing the culture medium after ∼24 h. We found that, after ∼48 h of uninterrupted transfection, the break-in (basal) and maximum current amplitudes were both markedly reduced in WT- compared with CiVSP C363S–transfected cells, resulting in many cells having no detectable endogenous TRPM7 currents (Fig. S1). We therefore conducted our experiments using moderate transfection conditions by removing the transfection reagent after ∼24 h.

The murine TRPM7 coding sequence (18) and its Ser-1107 point mutants in GFP-tagged (in the pEGFP-C1 plasmid vector) or untagged version (in pcDNA3) were transfected at 2.5 μg/well in 6 well-plates, and recordings were made 2–3 days after transfection. For co-transfection, 2.5 μg of TRPM7-pcDNA3 (WT or Ser-1107 point mutants) and 0.6–0.8 μg of WT Ci-VSP plasmid were used per well, and the majority of recordings were made 2 days after transfection. Occasionally, recordings were made 3 days after transfecting with VSP with similar results.

### Site-directed mutagenesis

Serine 1107 (41) of murine TRPM7 in pEGFP-C1 and pcDNA3 vectors was mutagenized to alanine, glutamate, glutamine, cysteine, threonine, lysine, or arginine using the QuikChange II XL kit (Promega, Madison, WI). All mutations were verified by DNA sequencing (Retrogen, San Diego, CA).

### Patch clamp electrophysiology

Whole-cell patch clamp recordings were performed as described previously (6, 20). Pipettes were manufactured from patch capillary glass (Warner Instruments, Hamden, CT) using a DMZ Universal (Zeitz Instruments, Martinsried, Germany) or P-1000 micropipette puller (Sutter Instrument, Novato, CA) and had resistances of ∼2–4 megaohm. Currents were recorded with an EPC10 patch clamp amplifier and Patchmaster software (HEKA Elektronik, Lambrecht, Germany). On the day of the experiment, cells were lifted off the polystyrene culture plate and transferred to the recording chamber mounted on the mechanical stage of an inverted microscope equipped with epifluorescence (Nikon). Successfully transfected cells were identified by their EGFP fluorescence and selected for patch clamp recording. In several experiments, we also recorded from nonfluorescent cells for comparison to rule out nonspecific transfection effects (Fig. 6*F*). For recording endogenous TRPM7 currents, the internal (pipette) solution contained 106 mm l-glutamic acid, 8 mm NaCl, 5 mm CsF, 10 mm HEDTA, and 10 mm HEPES acid (pH adjusted to 7.3 with CsOH). For the experiments described in Fig. 3, the pH of this solution was brought to 6.5 or 8.2 on the day of the experiment. For preparing solutions containing 10 μm and 150 μm free Mg^2+^, glutamic acid and HEDTA concentrations were lowered to 101 mm and 8 mm, respectively. For recordings of overexpressed mTRPM7 and its point mutants (Figs. 7 and 8), the internal solution contained 112 mm glutamic acid, 8 mm NaCl, 5 mm CsF, 12 mm EGTA, 10 mm HEPES acid, and 0.09 mm CaCl_2_ (pH 7.3 with CsOH). In all experiments (except Fig. 6), the external (bath) solution contained 2 mm CaCl_2_, 4.5 mm KCl, 140 mm sodium aspartate, 10 mm HEPES-Na^+^, 3 mm CsCl, and 0.5 mm glucose (pH 7.3). 1.0 m MgCl_2_ and CaCl_2_ standard solutions (Sigma-Aldrich, St. Louis, MO) were used for buffer preparation. 20 mm sodium aspartate was replaced with 20 mm sodium propionate in the standard 2 mm Ca^2+^ buffer to perform the experiments described in Fig. 4.

For Fig. 6, divalent cation–free (DVF) external solution was used, consisting of 140 mm aspartic acid, 6 mm HEDTA, and 10 mm HEPES (pH 7.3 with CsOH) or 140 mm sodium aspartate, 6 mm HEDTA, and 10 mm HEPES (pH 7.3 with NaOH). Deionized water (Nanopure, Barnstead, UK) was used in preparation of recording solutions. Osmolalities were measured with a freezing point depression osmometer (Precision Systems, Dubuque, IA) and adjusted by adding D-mannitol. Free Mg^2+^ concentrations were calculated with Webmaxc software.

Endogenous HEK cell and heterologous TRPM7 channel currents (20) were evoked by applying 211-ms command voltage ramps ranging from −100 to +85 mV every 2.5 s. In the experiments shown in Figs. 4, 5, 7, and 8, −85 to +85 mV voltage ramps of the same duration and frequency were used, except for a few recordings where 2 s gaps were used (Fig. 7, legend). Current traces were filtered at 2.9 kHz and digitized at 5 kHz. Data acquired with Patchmaster were saved on the hard drive of a personal computer for subsequent analysis. For recording monovalent TRPM7 currents in DVF solutions, voltage ramps from −100 to +20 mV were used (Fig. 6). In some cells, currents continued to rise slowly after 6 min at ∼1.5% per min; these small increases were ignored and 6 min magnitude value taken as the maximum (see Fig. 2).

Occasionally, endogenous I_TRPM7_ amplitudes recovered again after initial inhibition by 150 μm Mg^2+^ (data not shown). This rebound phenomenon was only observed in C363S- but not WT VSP–transfected cells. Such cells were excluded from our analysis.

Membrane potentials of untransfected HEK293 cells were measured using the perforated patch technique, essentially as described previously (7). Briefly, an aliquot of amphotericin B (Sigma) prepared in DMSO was dissolved in the internal solution containing 55 mm KCl, 50 mm K_2_SO_4_, 7 mm MgCl_2_, 1 mm CaCl_2_, and 10 HEPES (pH 7.3) to a final concentration of ∼240 μg/ml. The external solution contained 77 mm sodium aspartate, 62.5 mm NaCl, 2 mm CaCl_2_, 4.5 mm KCl, 10 mm HEPES–sodium, and 0.5 mm glucose (pH 7.3). Membrane potentials were recorded in current clamp mode continuously for ∼1–6 min (10-kHz sampling rate) with I_membrane_ = 0 after access resistances dropped to 20 MOhm or lower. For recording TRPM7 currents (Fig. 4*A*), the perforated patch solution contained Cs^+^ instead of K^+^. 7 mm Mg^2+^ present in this solution was sufficient to inhibit TRPM7 currents when whole-cell break-in occurred spontaneously. All experiments were performed at room temperature (∼25 °C).

### Molecular structure presentation

A partial structural model for mouse TRPM7 was developed using homology modeling methods implemented by Phyre2 (74, 75). The α kinase domain, as well as regions of TRPM7 primary structure for which satisfactory structural homologs could not be identified, are not included in our model. To examine the structural consequences of mutations in the truncated TRPM7 model, it was energy-minimized using the GROMOS force field, and the amino acid residue at position 1107 in the sequence was modified to alternate residues using Swiss PDB Viewer utilities. The resultant structures were again energy-minimized. Threading energies at each amino acid residue of these resulting structures were calculated. For each mutant protein, threading energies for residues within 8 Å of position 1107 were used to estimate the energetic consequences of the respective mutation. The solvent-accessible surfaces of each residue in the native and mutated TRPM7 structural models were also determined using Swiss PDB Viewer. Energy calculations were performed using the GROMOS96 43B1 parameter set, without reaction field, as implemented in Swiss PDB Viewer (76). In these calculations, solvent was not explicitly included.

### Chemicals and data presentation

l-glutamic acid, HEPES, EGTA, and HEDTA were from Acros Organics (Geel, Belgium). D-mannitol, NaOH, and DMSO were from Fisher Scientific. All other salts and 2-APB were purchased from Sigma-Aldrich. 200 mm stock solution of 2-APB was prepared in DMSO and diluted in the external recording solution on the day of the experiment. Analysis was done by comparing data collected from groups of cells transfected with WT, inactive VSP mutants, and/or mTRPM7 constructs and presented as scatterplots and bar graphs of means ± S.E. Patch clamp data curve fitting and graphing were performed using Origin versions 8, 8.6, and 2016 (OriginLab, Northampton, MA). Numbers of cells in graphs are given in parentheses. Statistical differences were compared using analysis of variance, Student's *t* test, and Tukey's multiple comparisons test.

## Author contributions

T. Z., K. B. W., P. B., and J. A. K. data curation; T. Z., P. B., M. M., and J. A. K. investigation; T. Z., P. B., G. M. A., and J. A. K. methodology; K. B. W., P. B., and J. A. K. formal analysis; G. M. A. software; G. M. A. visualization; M. M. resources; J. A. K. conceptualization; J. A. K. supervision; J. A. K. funding acquisition; J. A. K. validation; J. A. K. writing-original draft; J. A. K. project administration; J. A. K. writing-review and editing.

## Supplementary Material

Supporting Information

## Footnotes

3 The abbreviations used are:

TRP: transient receptor potential
PI(4,5)P_2_: phosphatidyl inositol bisphosphate
VSP: voltage-sensing phospholipid phosphatase
2-APB: 2-aminoethyl diphenyl borinate
HEK: human embryonic kidney
I–V: current–voltage
GOF: gain-of-function
EGFP: enhanced GFP
HEDTA: *N*-(2-hydroxyethyl)ethylenediaminetriacetic acid
DVF: divalent cation–free
CiVSP: *Ciona intestinalis* VSP
DrVSP: *Danio rerio* VSP.
