# Supplementary material for: Depletion of plasma membrane–associated phosphoinositides mimics inhibition of TRPM7 channels by cytosolic Mg2+, spermine, and pH
Source: J Biol Chem. 2018 Oct 10;293(47):18151–67. doi: 10.1074/jbc.RA118.004066 (PMC6254349; doi:10.1074/jbc.RA118.004066)
Supplement: Supporting Information [file supp_293_47_18151__index.html]

Depletion of plasma membrane-associated phosphoinositides mimics inhibition of TRPM7 channels by cytosolic Mg2+, spermine and pH — PIP2 depletion mimics TRPM7 inhibition by Mg2+ and pH — Depletion of plasma membrane–associated phosphoinositides mimics inhibition of TRPM7 channels by cytosolic Mg2+, spermine, and pH — PIP2 depletion mimics TRPM7 inhibition by Mg2+ and pH — Supporting Information 

# Depletion of plasma membrane–associated phosphoinositides mimics inhibition of TRPM7 channels by cytosolic Mg2+, spermine, and pH

## Supporting Information

- Suppl. Figure S1 - Figure
- Suppl. Figure S2 - Suppl. Fig. 2
