# Supplementary material for: Depletion of plasma membrane–associated phosphoinositides mimics inhibition of TRPM7 channels by cytosolic Mg2+, spermine, and pH
Source: J Biol Chem. 2018 Oct 10;293(47):18151–67. doi: 10.1074/jbc.RA118.004066 (PMC6254349; doi:10.1074/jbc.RA118.004066)
Supplement: Supporting Information [file supp_RA118.004066_138290_2_supp_213782_pg30g0.pdf]

Supporting Information

Depletion of plasma membrane-associated phosphoinositides mimics inhibition of TRPM7 channels by cytosolic Mg^{2+}, spermine and pH

Tetyana Zhelay[1], Krystyna B. Wieczerzak[1 ¶], Pavani Beesetty[1¶], Gerald M. Alter[2], Masayuki Matsushita[3] and J. Ashot Kozak[1]*

[1]Department of Neuroscience, Cell Biology and Physiology, Wright State University, Dayton, OH 45435, USA; [2]Department of Biochemistry and Molecular Biology, Wright State University, Dayton, OH 45435, [3]Department of Molecular and Cellular Physiology, Graduate School of Medicine, University of the Ryukyus, Okinawa 903-0215, Japan

¶ these authors contributed equally to this work
*corresponding author

Content

Figure S1: Effect of high levels of WT and C363S VSP expression on break-in (I_0) and maximum (I_{max}) current amplitudes.

Figure S2: Graph in Fig. 8D in full scale.

Figure S1

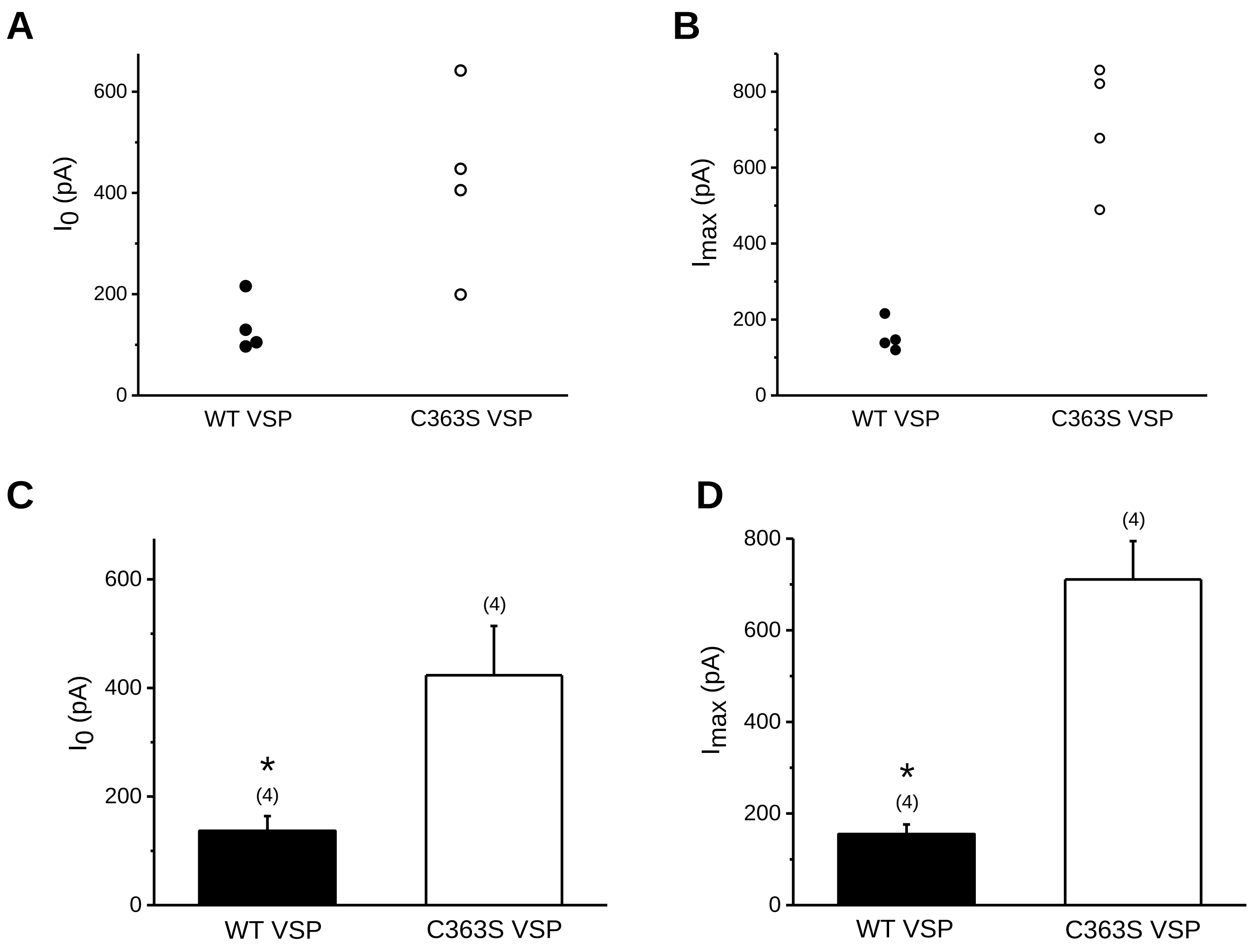

Figure S1. Effect of high levels of WT and C363S VSP expression on break-in (I_0) and maximum (I_{max}) current amplitudes. A, B. Scatter graph of I_0 and I_{max} amplitudes in WT (filled symbols) and C363S-expressing (empty symbols) cells. C, D. Summary of data shown in A and B. For these measurements HEK cells were incubated in the transfection mix containing VSP plasmids for 2 days without washout. Voltage ramps were followed by a 4 s duration step to +30 mV. *$p<0.05$, Student's pairwise t test. Internal free [Mg^{2+}] ≈ 100 nM.
