# Supplementary figures and images for: Depletion of plasma membrane–associated phosphoinositides mimics inhibition of TRPM7 channels by cytosolic Mg2+, spermine, and pH
Source: J Biol Chem. 2018 Oct 10;293(47):18151–67. doi: 10.1074/jbc.RA118.004066 (PMC6254349; doi:10.1074/jbc.RA118.004066)

Figure S2

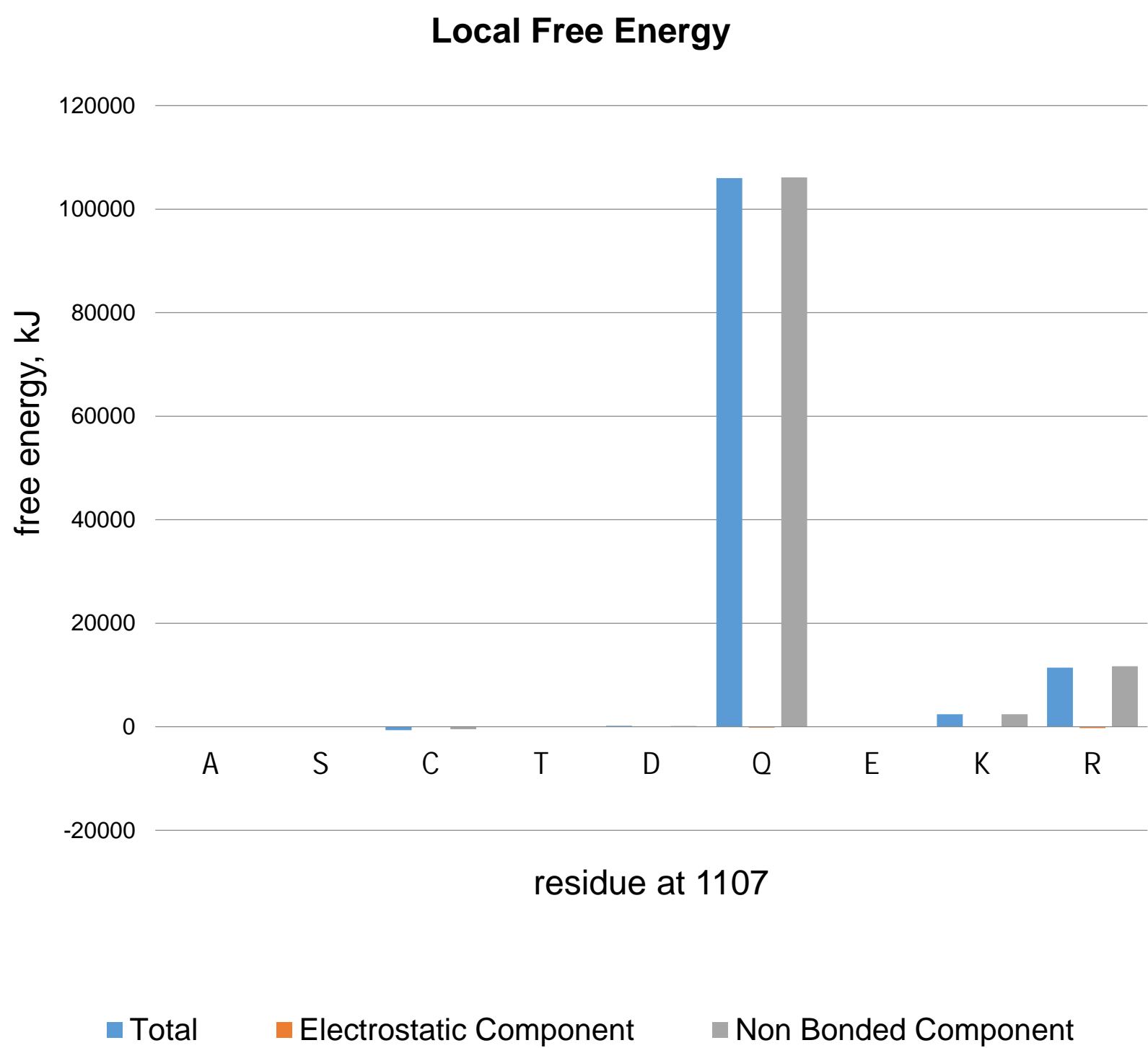

Figure S2. Graph in Fig. 8D in full scale.

Supplement: Supporting Information [file supp_RA118.004066_138290_2_supp_213790_pg1h0f.pdf]
